# Feedback damping of a microcantilever at room temperature to the minimum vibration amplitude limited by the noise level

**DOI:** 10.1038/srep27843

**Published:** 2016-06-17

**Authors:** Y. Kawamura, R. Kanegae

**Affiliations:** 1 Department of Intelligent Mechanical Engineering, Faculty of Engineering, Fukuoka Institute of Technology, 3-30-1 Wajirohigashi, Higashiku, Fukuoka, 811-0295, Japan

## Abstract

Cooling the vibration amplitude of a microcantilever as low as possible is important to improve the sensitivity and resolutions of various types of scanning type microscopes and sensors making use of it. When the vibration amplitude is controlled to be smaller using a feed back control system, it is known that the obtainable minimum amplitude of the vibration is limited by the floor noise level of the detection system. In this study, we demonstrated that the amplitude of the thermal vibration of a microcantilever was suppressed to be about 0.15 pmHz^−1/2^, which is the same value with the floor noise level, without the assistance of external cryogenic cooling. We think that one of the reason why we could reach the smaller amplitude at room temperature is due to stiffer spring constant of the lever, which leads to higher natural frequency and consequently lower floor noise level. The other reason is considered to be due to the increase in the laser power for the diagnostics, which lead to the decrease in the signal to noise ratio determined by the optical shot noise.

Cooling the vibration amplitude of micromechanical resonators as low as possible has been a common interest of physics and engineering from a wide range of scientific perspectives^1^,^2^,^3^,^4^. There have been two main approaches followed.

One is the regime represented by a “nano”-resonator with relatively high natural frequencies (several tens of MHz–several GHz) and quantum methods for the detection of the displacement, such as a quantum bit spectroscopy. The purpose in this regime is to decrease the amplitude of the vibration sufficient to reach the quantum ground state^5^,^6^,^7^,^8^,^9^,^10^,^11^, and therefore the physical interests.

The other is the regime represented by a “microcantilever” with relatively low natural frequencies (several kHz–several tens of kHz) and classical methods of detection, such as an optical interferometry^11^,^12^,^13^,^14^,^15^,^16^,^17^,^18^,^19^,^20^,^21^. In this case, the minimum for the quantum number obtained was large at 2.1 × 10^4^, which was achieved aided by cryogenic cooling of the resonator to several Kelvins^15^.

The objective of the former regime was mainly the physical demonstration of the existence of the quantum zero-energy point and its application in studies on basic physical phenomena. For the latter regime, the concern was mainly the applications of the silicon micro-cantilever to the more sensor detecting technologies^18^,^19^,^20^,^21^.

The study in this paper belongs to the latter regime. we demonstrated that the amplitude of the power spectrum density of the thermal vibration of a microcantilever was suppressed to be about 0.02 pm^2^/Hz, which is the same value with the floor noise level, without the assistance of external cryogenic cooling. This value is two orders of magnitude smaller than that of the previous work^15^, although the experiment was performed at room temperature, while the previous work was done at 4.2 K.

## Methods

**Figure 1:**
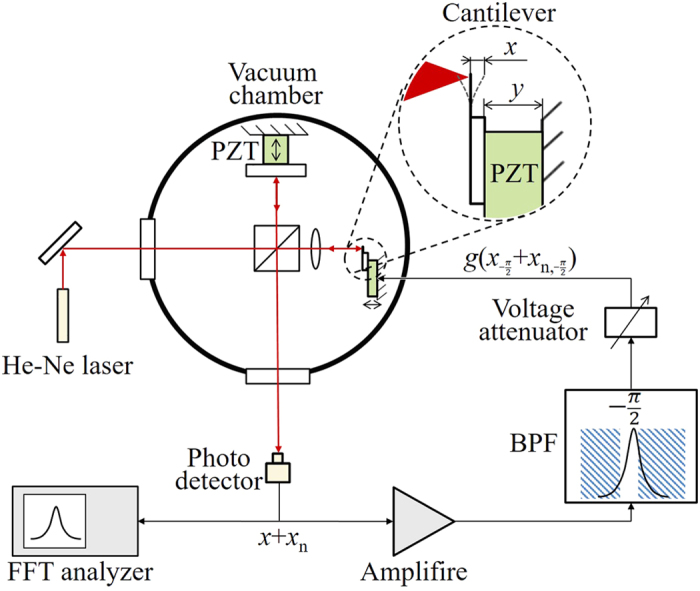
Experimental setup for feedback cooling of the thermal vibration of a micro-silicon cantilever. (PZT: piezoelectric actuator, BPF: band pass filter, FFT: fast Fourier transformer).

The experimental system (Fig. 1) employs a commercially available silicon micro-cantilever used as a resonator, with length, width, and thickness of 240 μm, 40 μm, and about 2.3 μm, respectively. The catalog value of the spring constant, *k*, is 2 N/m. The natural frequency of the microlever, *f*_0_, was measured at 80.8 kHz. The cantilever was mounted on a single layer piezoelectric actuator (PZT) to be able to change the amplitude relative to the inertial frame (*x* + *y* in Fig. 1). The amplitude of the actuator (*y*) is small enough compared with the amplitude of the cantilever (*x*) and neglected in this experiment. The system was placed in a vacuum chamber, which was evacuated down to 5 × 10^−3^ Pa using an oil diffusion vacuum pump.

The vibration amplitude of the silicon microlever (*x*) was measured by Michelson interferometry. A He-Ne laser of 1 mW was used as the light source of the interferometer. To be free of fluctuations of the interferometer, the operation point is set automatically at its most responsive point and controlled.

The output signal of the interferometer was passed through a band pass filter with a band width of about 10 kHz. The central frequency of the window range was adjusted to obtain the phase shift of *φ* = 90°.

To change the loop gain, (*g*) of the feedback control, the drive signal of the PZT actuator was changed by the voltage attenuator. The voltage attenuator was a capacitive type making use of the capacity of the PZT. *g* was defined as the loop gain, therefore it is defined as the ratio of the movement of the PZT to that of the cantilever.

The vibration control method used here is fundamentally the same as that used for normal mechanical systems including large-scale ones, such as high-rise buildings^22^,^23^. The whole experimental system was placed on an anti-vibration table having a natural frequency of about one second, and the resonator system of the silicon lever was placed on a rubber block in the vacuum chamber for greater vibrational isolation.

The output signal from the photodetector was recorded and processed using a Fourier transform spectrum analyzer. The quality factor of the resonance, *Q*, was measured to be around 1.0 × 10^5^. The *Q* was also measured for the same silicon cantilever with aluminum coating. It was only about 3000, which means that the aluminum coating operates as the dumping factor.

According to a mathematical analysis of feedback cooling with noise signal added to the feedback loop^4^,^15^, the power spectral density for the *actual* vibration amplitude is calculated to be
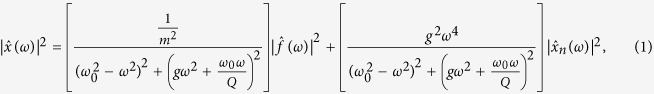
and the power spectral density for the *measured* vibration amplitude is calculated to be
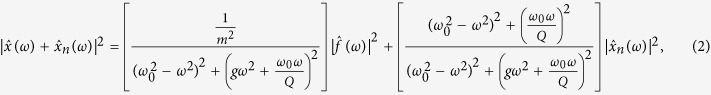
where 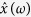 and 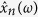 are the complex amplitude of vibration of the cantilever and the noise signal. 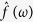 is the averaged Langevin force, which generates the thermal vibration in the micro cantilever. *ω*_0_ and *Q* are the natural angular frequency and the quality factor, respectively, of the fundamental vibration mode of the microlever, and *m* is the equivalent mass of the cantilever. *g* is the feedback loop gain. Here, “*actual*” means the real value of the amplitude, which does not include the noise signal of the detection system, and “*measured*” means the apparent value of the amplitude, which appears in the detection system including the noise signal. It is noted that the former is larger than the latter, and 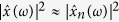 at the limit of large value of *g*.

## Results

**Figure 2:**
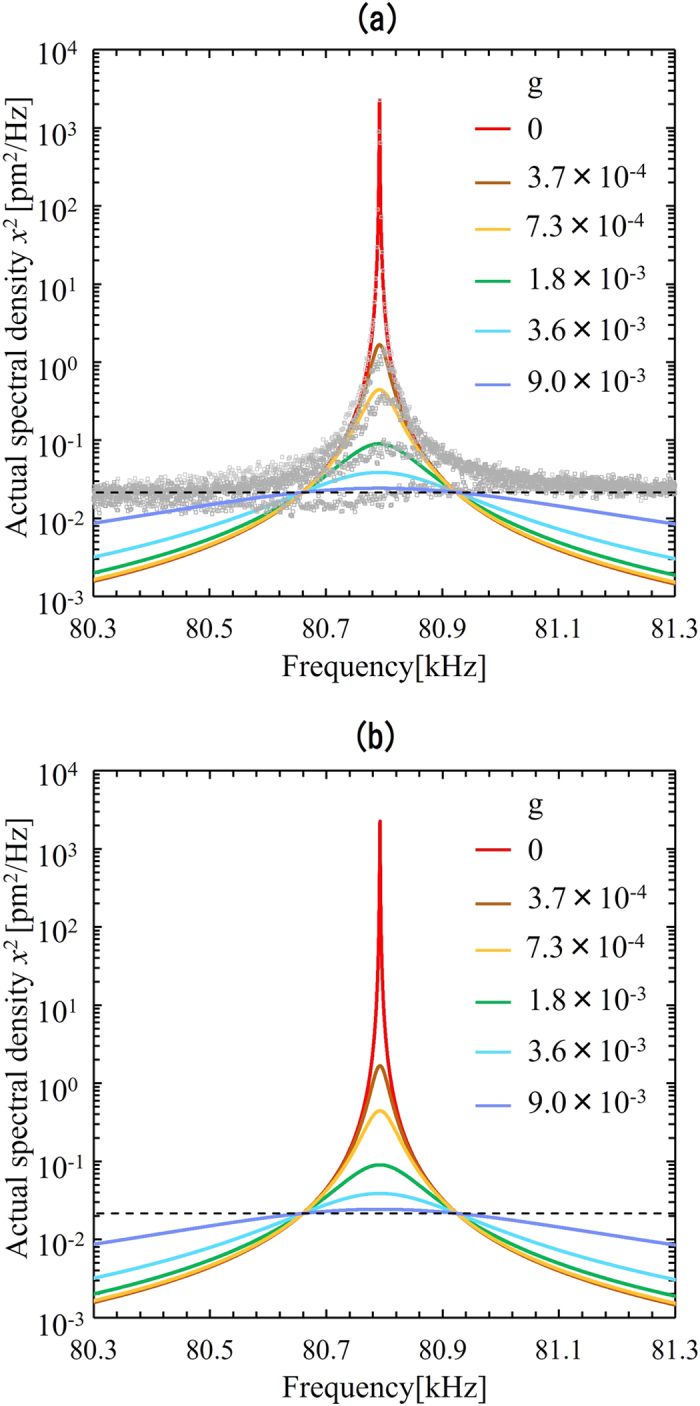
Power spectra of the vibration amplitude. (**a**) *Measured* power spectra of vibration amplitude of a micro-cantilever for various feedback loop gains. Solid lines are theoretical calculations obtained using Eq. (2). (**b**) *Actual* power spectrum of the vibration amplitudes of a micro-cantilever for various feedback loop gains. Solid lines are the theoretical calculations obtained by Eq. (1). Dotted line is the back ground noise level (*x*_n_).

Figure 2(a) presents the *measured* power spectrum ((*x* + *x*_n_)^2^) of the vibration of a micro-cantilever for various feedback loop gains. As the feedback gain increases, it decreases, and finally falls below that of floor noise level. The solid lines are the theoretical calculations for each feedback gain (see Eq. (2)). We find good agreement between the experimental results and the theoretical calculations for all values of the feedback gain.

The *actual* power spectrum (*x*^2^) of the vibration amplitudes, which cannot be detected directly, are given for various loop gains in Fig. 2(b); the solid lines are the theoretical calculations (see Eq. (1)). For small values of gain, the experimental results are in good agreements with the theoretical calculation, whereas discrepancies appear for larger values of the loop gains. We can see that the *actual* amplitude converges to the floor noise level, as loop gain increases. Therefore the obtainable minimum actual amplitude must be larger than the floor noise.

**Figure 3:**
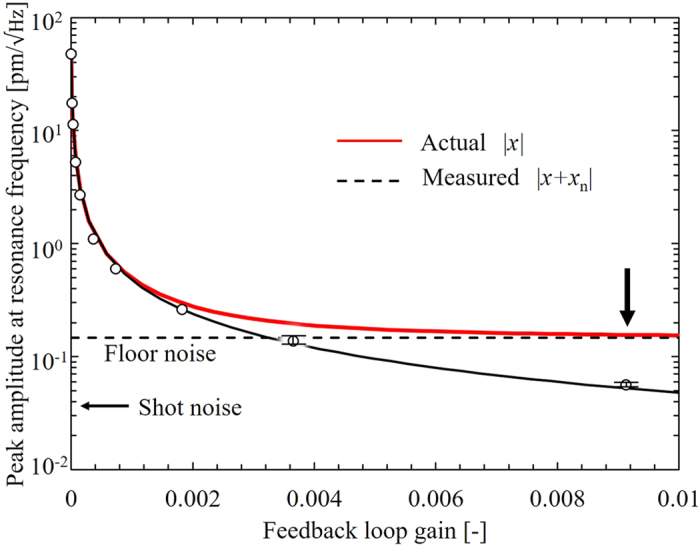
Amplitudes of the vibration at the natural frequency (*f*_0_) as a function of the feedback loop gain (*g*). Open circles are *measured* values. Red solid and black dashed lines are obtained from theoretical calculations for the *actual* and *measured* values, using Eqs (1) and (2), respectively. The horizontal black dashed line shows the limitation determined by the floor noise level of the detection system. The shot noise level due to the diagnostic laser is indicated by the horizontal black arrow. The position indicated by the vertical arrow on the red solid line at *g* = 9.0 × 10^−3^ shows the minimum *actual* vibration amplitude obtained.

The *actual* and the *measured* amplitude of the cantilever at the natural frequency are plotted in Fig. 3 as functions of the loop gain in comparison with the floor noise level and the amplitude for the shot noise due to the diagnostic laser. The position indicated by the vertical arrow on the red solid line at *g* = 9.0 × 10^−3^ shows the minimum *actual* vibration amplitude obtained. At this point, the power spectral density for the vibration amplitude is about 0.02 pm^2^ Hz^−1^, which is two orders of magnitude smaller than that of the previous work^15^, even though this experiment was performed at room temperature and the previous work was done at 4.2 K.

## Discussions

A theoretical value of the thermal vibration amplitude of the silicon lever was calculated to be 4.5 × 10^−11^ m (45 pm), assuming that energy *k*_B_*T/*2 is distributed as averaged potential energy; here *k*_B_ is the Boltzmann constant and *T* is the temperature of the surrounding system (300 K). In this calculation, the cantilever was modelled as a mass-spring oscillator with the mass attached to the tip of the lever. The equivalent mass was calculated to be 7.8 × 10^−12^ kg setting *k* = 2 N/m and *f*_0_ = 80.8 kHz. The measured amplitude of the thermal vibration was 58 pm, which was larger than the theoretical calculation of 45 pm. Using the thermal and optical characteristics of Si, a heat transfer analysis gives a temperature rise estimate of about 10 K, by which the above discrepancy could not be fully explained. In this estimation, we assumed that the absorbed energy of the diagnostic laser beam was 0.60 mW considering that the absorption coefficient of silicon at visible wavelengths is about 60% (reflectivity: 40%). The dynamic vibration mode profile of the cantilever was simplified to that of the static bending profile of the cantilever, which may be the reason for the discrepancy.

The main features of this experiment compared with that of a previous study^15^ are the use of a micro-cantilever with large spring constant (relative ratio: about 23,000), consequently the natural resonance frequency was increased about 31 times, and the use of a relatively high power for the diagnostic laser (relative value: about 10,000).

The signal to noise ratio determined by the power of the diagnostic laser is inversely proportional to the root of the laser power, it is one of the reason why we could decrease the floor noise revel comparing to the previous works^15^. Moreover, the increase in the spring constant increases the natural vibration frequency, which consequently decreases the mechanical vibration noise. These are considered to be the reasons why we could attain the minimum amplitude of a microcantilever vibration determined by a floor noise at room temperature.

## Conclusions

In this study, we demonstrated that the amplitude of the thermal vibration of a microcantilever was suppressed to be about 0.15 pmHz^−1/2^, which is the same value with the floor noise level, without the assistance of external cryogenic cooling. We think that one of the reason why we could reach the smaller amplitude at room temperature is due to stiffer spring constant of the lever, which leads to higher natural frequency and consequently lower floor noise level. The other reason is considered to be due to the increase in the laser power for the diagnostics, which lead to the decrease in the signal to noise ratio determined by the optical shot noise.

## Additional Information

**How to cite this article**: Kawamura, Y. and Kanegae, R. Feedback damping of a microcantilever at room temperature to the minimum vibration amplitude limited by the noise level. *Sci. Rep.* **6**, 27843; doi: 10.1038/srep27843 (2016).

## References

[b1] Wilson-Rae I., Zeller P. & Imamoglu A. Laser cooling of a nanomechanical resonator mode to its quantum ground state. Phys. Rev., Lett. 92, 075507-1–075507-4 (2004). 14995872 10.1103/PhysRevLett.92.075507

[b2] Marshall W., Simon C., Penrose R. & Bouweester D. Towards quantum superposition of a mirror. Phys. Rev. Lett. 91, 130401-1–130401-4 (2003). 14525288 10.1103/PhysRevLett.91.130401

[b3] Schwab K. C. & Roukes M. L. Putting mechanics into quantum mechanics. Physics Today 58, 36–42 (2005).

[b4] Aspelmeyer M., Kippenberg T. J. & Marquardt F. Cavity optomechanics. *Rev. Mod. Phys*. 86, 1391–1452 (2014).

[b5] LaHaye M. D., Buu O., Camarota B. & Schwab K. C. Approaching the Quantum Limit of a Nanomechanical Resonator. Science 304, 74–77 (2004). 15064412 10.1126/science.1094419

[b6] Knobel R. G. & Cleland A. N. Nanometre-scale displacement sensing using a single electron transistor. Nature 424, 291–293 (2003). 12867975 10.1038/nature01773

[b7] Sazonova V. . A tunable carbon nanotube electromechanical oscillator. Nature 431, 284–287 (2004). 15372026 10.1038/nature02905

[b8] O’Connell A. D. . Quantum ground state and single-phonon control of a mechanical resonator. Nature 464, 697–703 (2010). 20237473 10.1038/nature08967

[b9] Poot M., Fong K. Y. & Tang H. X. Deep feedback-stabilized parametric squeezing in an optoelectromechanical system. New J. Phys. 17, 1–12 043056 (2015).

[b10] Poot M., Fong K. Y. & Tang H. X. Classical non-Gaussian state preparation through squeezing in an optoelectromechanical resonator. Phys. Rev. A 90, 063809-1–3 (2014).

[b11] Vogel M., Mooser C., Karrai K. & Warburton R. J. Optically tunable mechanics of microlevers, Apply. Phys. Lett. 83, 829–830 (2003).

[b12] Metzer C. H. & Karria K. Cavity cooling of a microlever. Nature 432, 1002–1005 (2004). 15616555 10.1038/nature03118

[b13] Kleckner D. & Bouwmeester D. Sub-kelvin optical cooling of a micromechanical resonator. Nature 444, 75–78 (2006). 17080086 10.1038/nature05231

[b14] Kleckner D. . High finess opto-mechanical cavity with a mobile thirty-micron size mirror. Phys. Rev. Lett. 96, 173901-1–173901-4 (2006). 16712296 10.1103/PhysRevLett.96.173901

[b15] Poggio M., Degen C. L., Mamin H. J. & Rugar D. “Feedback cooling of a cantilever’s fundamental mode below 5 mK. Physical Review Letters 99, 017201 (2007). 17678185 10.1103/PhysRevLett.99.017201

[b16] Cohadon P. F., Heidmann A. & Pinard, M. Cooling of a Mirror by Radiation Pressure. Physical Rev. Lett. 83, 3174–3177 (1999).

[b17] Arcizet O. . Radiation-pressure cooling and optomechanical instability of a micromirror. Nature 444, 71–74 (2006). 17080085 10.1038/nature05244

[b18] Mamin H. J. & Rugar D. Sub-attonewton force detection at millikelvin temperatures. App. Phys. Lett. 79, 3358–3360 (2001).

[b19] Stowe T. D. . Attonewton force detection using ultrathin silicon cantilevers. Appl. Phys. Lett. 71, 288–290 (1997).

[b20] Sidles J. A. . Magnetic resonance force microscopy. Rev. Mod. Phys. 67, 249–265 (1995).

[b21] Binning G., Quate C. F. & Gerber C. Atomic force microscope. Phys. Rev. Lett. 56, 930–933 (1986). 10033323 10.1103/PhysRevLett.56.930

[b22] Nagashima I. . Performance of hybrid mass damper system applied to a 36 storey high-rise building. The journal of the international association for earthquake engineering 30, 1615–1638 (2001).

[b23] Abiru H. . “Tuned active dampers installed in the Yokohama Landmark Tower”, The Japan society of mechanical engineers C37, 450–455 (1994).

